# *Lactobacillus*-Derived Bioactive Metabolites for the Regulation of Periodontal Health: Evidences to Clinical Setting

**DOI:** 10.3390/molecules25092088

**Published:** 2020-04-29

**Authors:** Benso Sulijaya, Naoki Takahashi, Kazuhisa Yamazaki

**Affiliations:** 1 Department of Periodontology, Faculty of Dentistry, Universitas Indonesia, Jakarta 10430, Indonesia; bensosulijaya@gmail.com or; 2 Division of Periodontology, Department of Oral Biological Science, Niigata University Graduate School of Medical and Dental Sciences, Niigata 951-8514, Japan; takahashi-n@dent.niigata-u.ac.jp; 3 Research Unit for Oral-Systemic Connection, Division of Oral Science for Health Promotion, Niigata University Graduate School of Medical and Dental Sciences, Niigata 951-8514, Japan

**Keywords:** fatty acid, metabolite, gut bacteria, periodontal disease

## Abstract

Background: Gut microbiota plays a pivotal role in regulating host metabolism that affects the systemic health. To date, several studies have confirmed the fact that microbiota interacts with host, modulating immunity, controlling the homeostasis environment, and maintaining systemic condition. Recent studies have focused on the protective function of poly unsaturated fatty acids, 10-oxo-trans-11-oxadecenoic acid (KetoC) and 10-hydroxy-*cis*-12-octadecenoic acid (HYA), generated by gut microbiota on periodontal disease. Nevertheless, the mechanism remains unclear as investigations are limited to in vivo and in vitro studies. In this present review, we found that the administration of metabolites, KetoC and HYA, by a probiotic gut microbiota *Lactobacillus plantarum* from linoleic acid is found to inhibit the oxidation process, possess an antimicrobial function, and prevent the inflammation. These findings suggest the promising use of functional lipids for human health. Conclusion: Protective modalities of bioactive metabolites may support periodontal therapy by suppressing bacterial dysbiosis and regulating periodontal homeostasis in the clinical setting.

## 1. Introduction

A number of studies have confirmed the fact that commensal microbiota interacts with the host, regulating the bioconversion of nutrients and detoxification, modulating immunity, controlling the homeostasis environment, thereby maintaining systemic health [1]. For example, the depletion of gut microbiota in mice shows a large defect of intestine-associated lymph tissues, a lower abundance of intestinal secretory IgA antibodies, and smaller mesenteric lymph nodes [2,3]. Moreover, it is well documented that intestinal bacterial metabolites such as acetate, butyrate, and propionate are the source of energy for epithelial cells, inhibitors of inflammation, and modulators of insulin secretion [4,5,6]. 

On the other hand, accumulating evidence also shows that the dynamics and function of indigenous microbiota can be influenced by many factors, including genetics, diet, age, drugs, and contaminants [1]. In obese patients, a high intake of fats and sugar may alter the gut microbial composition and reduces its diversity [7,8]. This alteration causes a long-term imbalance of energy intake regulation and metabolism activities in the gut, thereby developing obesity [9]. This idea enforces the regulation of gut microbiota influenced through dietary intake. Furthermore, oral and gut microbiota have been associated with the development of atherosclerotic cardiovascular disease through microbial trimethylamine synthesis [10]. Besides metabolic alteration of the bacteria, it is critical to point out that increased abundance of the pathogen in the oral cavity in periodontitis patient might reach the circulation and contribute to low-grade inflammation, thereby establishing an atherosclerotic cardiovascular disease [10] while the route from the oral cavity to the systemic circulation is not clarified.

A specific diet affects oral and gut microbiota composition. A high intake of carbohydrates was reported to have a higher abundance of acidogenic and aciduric bacteria such as *Lactobacilli* and *Streptococcus mutans* in the oral cavity [11]. Besides, fatty acid and vitamin supplementation show a positive association with *Betaproteobacteria* and *Fusobacteria*, while vitamin C intake correlates with a high abundance of *Fusobacteria*, *Leptotrichiaceae,* and *Lachnospiraceae* [11]. Long-term consumption of alcohol and tobacco leads to a reduction of bacterial richness, including *Neisseria, Fusobacteria, Granulicatella, Peptostresptococcus,* and *Gemella*, which relates to oral disease condition [12]. In severe periodontitis patients, 10^9^ CFU/mL of *Porphyromonas gingivalis* is swallowed with saliva to the intestine and induces inflammatory reactions [13]. Moreover, several studies have testified the association between periodontitis and inflammatory bowel disease, possibly through oral-gut dysbiosis and epithelial barrier function impairment [14,15,16,17,18]. With respect to the treatment of periodontitis, the adjunctive use of nutrition to scaling and root planing displayed beneficial outcomes [19,20]. These findings suggest that dietary intake and nutrition affect not only the local but also systemic homeostasis.

Studies have now started to focus on the beneficial function of specific bacterial metabolites for reducing disease risks. It is well documented the effect of poly unsaturated fatty acid (PUFA) generated by gut microbiota on periodontal disease [19,20,21,22,23,24,25,26,27,28,29]. Moreover, the administration of conjugated linoleic acid (CLA) catalyzed by *Lactobacillus plantarum* from linoleic acid is found to inhibit the initiation of mice skin carcinogenesis [30], rats tumorigenesis [31], and anti-inflammatory effect [32,33]. These findings suggest the promising use of functional lipids for human health. Therefore, in this paper, we aimed to critically review and highlight the generation and protective functions of metabolites generated by *Lactobacillus* with regard to further application in the management of periodontal disease.

## 2. *Lactobacillus*-Derived Bioactive Metabolites 

### 2.1. KetoC and HYA: Generation of Bioactive Metabolites by Lactobacillus

Intestinal microbiota regulates the saturation process of PUFA from dietary fat as a detoxifying mechanism [34]. Fatty acids can be transformed in many ways, for example, by elongation, insertion or removal of double bonds, or covalent binding proteins [35]. In addition, *L. plantarum* has been reported for its potential to convert linoleic acid (LA) to CLA [36]. In addition, 120 mg/mL LA can be converted to 40 mg/mL CLA by *L. plantarum* in 108 h [36]. The washed (resting) cells of lactic acid bacteria were used as catalysts, which can help to avoid the inhibitory effects of fatty acids (substrates) on cell growth during the process, thus enabling reactions with high substrate concentrations [37]. 

**Figure 1:**
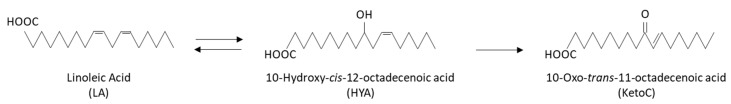
Chemical structure of these metabolites [26]. *Lactobacillus plantarum* converts LA to various metabolites (HYA and KetoC) through saturation process. HYA has a hydroxy-group, while KetoC has an oxo-group.

Based on the molecular and chemical structures, metabolites generated by *Lactobacillus* through polyunsaturated fatty acid (PUFA) process were 10-hydroxy-*cis*-12-octadecenoic acid (HYA), 10-hydroxy-octadecenoic acid (HYB), 10-hydroxy-*trans*-11-octadecenoic acid (HYC), 10-oxo-cis-12-octadecenoic acid (KetoA), 10-oxo-octadecanoic acid (KetoB), and 10-oxo-*trans*-11-octadecenoic acid (KetoC) [29]. Among these metabolites, KetoC and HYA have been shown to have positive effects on homeostasis. KetoC is recognized as a fatty acid with oxo-group in the strain number 10 and a double-bond in strain 11; while, HYA has hydroxy-group in the strain number 10 and cis at 12th of its structure (Figure 1) [24]. Beneficial effects of KetoC and HYA are summarized in Table 1.

### 2.2. Beneficial Functions of KetoC and HYA in the Physiological and Pathological Processes 

#### 2.2.1. Anti-Inflammatory Function

Modulating the inflammation becomes a treatment strategy for periodontitis [20]. Related to this approach, KetoC exerts anti-inflammatory function via Mitogen-activated protein kinase (MAPK) and NFκB signaling in macrophages induced with bacterial lipopolysaccharide (LPS) [27]. KetoC prevents Extracellular signal-regulated kinase (ERK) phosphorylation induced by LPS in microglial cells [39]. Further, 5 μM/L KetoC is found to partially inhibit translocation of NFκB p65 to the nucleus by binding to G-protein coupled receptor (GPR)120 in macrophages stimulated with *P. gingivalis* LPS [22]. KetoC inhibited the production of IL-6, IL-1β, and TNFα. Moreover, the suppression toward TNFα was in a dose-dependent manner, which explains the direct action of KetoC. Hence, a higher concentration of KetoC (50 μM/L) demonstrated a cytotoxic activity to macrophages [22].

GPRs, also have been identified as a free fatty acid receptor (FFAR), have been investigated for its physiological functions, e.g., hormone secretion, adipocyte differentiation, anti-inflammatory effect, and neuronal regulation [42]. For example, GPR40/FFAR1 is abundantly expressed in pancreatic insulin-producing β cells and the intestine, thereby associating with the development of obesity and diabetes [43]. While GPR120/FFAR4 is well-detected in many tissues and cell types, including the intestine, pancreas, adipocytes, and immune cells [44]. GPR41/FFAR3 is distributed in adipose tissue, intestine, and the peripheral nervous system [45,46]. Expressed in various tissues, accumulating studies have suggested that short-chain fatty acids (SCFAs) may activate GPR41/FFAR3 and GPR43/FFAR2, while medium-chain and long-chain FAs may stimulate GPR40/FFAR1 and GPR120/FFAR4 [44,47,48]. Based on the number of carbons, both HYA and KetoC are classified to medium- to long-chain FAs (Figure 1). Further, HYA augments the expression of GPR40 and GPR43 in Caco-2 cells [24], while KetoC action depends on the existence of GPR120 in macrophages [22]. Then, to clarify the interaction between metabolites and their receptors, the specific antagonist was used accordingly, such as GW1100 for GPR40 and AH7614 for GPR12 [22,23]. To the fact that these beneficial effects on physiological processes are considered to regulate energy and immune homeostasis, activation of GPR serves as a potential therapeutic target for energy metabolism disorders and immune-related disease [48]. Altogether, those metabolites work in the cells by binding to GPRs.

Regarding the signaling pathway regulating the inflammation, HYA is found to decrease TNFR2 expression in Dextran sulfate sodium (DSS)-induced colitis mice [24]. In respect to this, the upregulation of TNFR2 subsequently reduced the ratio of phospho-IκBα/IκBα and NFκB p65, thereby decreasing proinflammatory cytokine production. HYA decreases local inflammatory cytokine mRNA level (*IL-1β, TNFα*, and *IL-6*) in gingival tissue in vivo with a tendency of alveolar bone loss suppression [23]. HYA induces ERK phosphorylation; on the other hand, the blockade of the MEK-ERK signaling deteriorates the HYA-GPR40 anti-inflammatory action [24].

CLA isomer t10,c12 was found to reduce the expression of pro-inflammatory cytokines (IL-1β and TNFα) in human astrocytes [49]. This implies that fatty acid may also affect neuro-inflammation by suppressing pro-inflammatory molecules in cultured astrocytes, suggesting its potential inhibition to Alzheimer’s disease (AD). In addition to this, neuro-inflammation disease and periodontitis may contribute one to another. Association has been confirmed between AD and periodontitis by the expression of inflammatory cytokines, suggesting that periodontitis may be related to the onset, progression, and aggravation of AD [50]. In a different way, serum IgG antibody levels to bacteria associated with periodontitis were noticed with an increasing incidence of AD onset/progression among participants with high serum antibody [51]. Several investigations clearly pointed to inflammation as an important factor in both periodontitis and Alzheimer’s disease [52]. 

#### 2.2.2. Antimicrobial Function

Administration of 5 μM/L KetoC inhibits *P. gingivalis* proliferation rate in vitro, while 15 mg/mL reduces alveolar bone destruction in periodontitis mice model [21]. Further, by using the LIVE/DEAD bacterial staining kit, this metabolite could suppress bacterial viability compared to negative control [21]. Compare to other fatty acids, for example, Docosahexaenoic acid (DHA) and Eicosapentaenoic acid (EPA), a low concentration of KetoC (5 μM/L) exhibits bacteriostatic and bactericidal activities within 24 h [21]. In contrast, the antibacterial effect of EPA and DHA was noticed in a relatively high concentration (100 μM/L) compared to KetoC [53]. The antimicrobial effect of fatty acid might be due to the unsaturated double-bond structure existed in KetoC [54]. A study compared the presence of carbon–carbon double bond by comparing the efficacy of KetoC and KetoB in the suppression of *P. gingivalis* [21]. The distinction between KetoC and KetoB is simply the double-bond structure in the chain; unlike KetoB, KetoC contains double-bond [35]. The results demonstrated that KetoC, but not KetoB, subdues the viability, proliferation rate, and Ct values of *P. gingivalis* [21].

Moreover, the investigation of several PUFAs has demonstrated that more significant antibacterial effects are related to the degrees of unsaturation [55]. Fatty acids (EPA and DHA) are known to affect the integrity of bacterial membrane by increasing its fluidity and permeability, thus allowing small molecules, for example, hydrogen ions, to penetrate further into the bacteria, which causes damage [55,56,57]. Gram-negative bacteria are more susceptible to fatty acid due to a hydrophobic surface on the outer membrane of bacteria [58]. In this matter, hydrophobic molecules like KetoC might be easily attached to the bacterial outer membrane, then act accordingly. Scanning electron microscopy analysis revealed a morphological alteration of *P. gingivalis* in the presence of fatty acids [59]. Another explanation for antimicrobial activity might be the formation of toxic lipid peroxidases generated by an oxidative process involving hydrogen peroxide and iron by fatty acids [55,60]. In all, the antimicrobial function of KetoC towards *P. gingivalis* is assumed to be mediated by its hydrophobic characteristic and carbon–carbon double bond structure [21,26].

#### 2.2.3. Effect on Epithelial Barrier Function 

Disruption of the gingival epithelial barrier by specific proteases and their penetration into underlying tissue generates periodontal tissue breakdown in periodontitis [61,62]. In terms of bacteria, levels of beneficial and harmful microorganisms may interact with epithelial cells generating the gingival barrier function [17]. Beneficial bacteria, *Lactobacillus species, Bifidobacterium,* and *Streptococcus gordonii*, positively regulate the barrier function through direct and indirect pathways [17]. For instance, beneficial bacteria may induce antimicrobial peptides through host immune cells response against barrier-disrupting pathogens [63,64,65]. Another way beneficial bacteria and their derivatives (e.g., HYA and KetoC) regulate epithelial barrier is by stimulating tight junction (TJ)-related gene expression. In addition to this, beneficial bacteria may also create a favorable microenvironment that reduces pathogens. Harmful periodontal pathogens, such as *P. gingivalis*, *Tannerella forsythia*, *A. actinomycetemcomitans,* and *Treponema denticola*, have negative impacts on the gingival barrier function. In addition to this, *P. gingivalis* has been involved in the pathogenesis of periodontitis due to its variety of virulence factors, including fimbriae [66,67], lipopolysaccharides [68,69], capsule [70,71], and proteases [72,73]. Therefore, gingival epithelial cells play crucial roles in the initiation and progression of periodontal diseases by acting as a physical barrier to periodontopathic bacteria [23].

The previous investigation documented that *P. gingivalis* degraded barrier function-related proteins, such as E-cadherin and β-catenin [23]. E-cadherin is an essential adhesion molecule for barrier formation by giving durable cell-cell contact between epithelial cells [74]. Meanwhile, β-catenin serves as a complex partner of E-cadherin and maintains the adherence junction complexes by connecting the cytoskeleton with E-cadherin [75]. Recent investigations have found a low level of E-cadherin expression in the gingival epithelium of periodontitis subjects, assuming E-cadherin as a key in the initiation of periodontal disease [76,77].

HYA treatment suppressed this degradation by promoting the proteolytic resistance of E-cadherin/β-catenin toward *P. gingivalis* in the gingival epithelial cells [23] and colorectal epithelial adenocarcinoma cells (Caco-2) [39], thereby reducing the inflammation and injury. An investigation reported that tight junction permeability (TER) and protein molecules (occluding, ZO-1, ZO-2, and claudin-3) were impaired by INF-γ and TNFα challenge in Caco-2 cells through the upregulation of IL-8, and then recovered by the pretreatment of HYA in a dose-dependent manner [39]. Moreover, the action of HYA depends on the expression of GPR40 in gingival epithelial cells [23]. 

#### 2.2.4. Anti-Oxidant Function and Other

Periodontitis has been associated with oxidative stress-related disease [40,78,79,80]. In particular, a study clearly showed that reactive oxygen species (ROS) induced by pathogenic bacteria generates intracellular signaling pathways and promotes proinflammatory cytokines production, such as IL-6 and TNFα in gingival epithelial cells [81]. Furthermore, the production of ROS activates c-Jun *N*-terminal Kinase (JNK) signaling, leading to an impairment of E-cadherin in the junctional epithelium of the periodontal apparatus [82]. These proofs suggest that over a generation of ROS may initiate periodontitis. Nowadays, a current strategy for host modulation therapy (HMT) in periodontitis is developed by regulating the inflammation and oxidative stress condition [20].

Proposed as an HMT agent, KetoC stimulates antioxidant-related gene expression in gingival epithelial cells [83], and human liver cells through the Nrf2-ARE signaling pathway [26]. Treatment with KetoC, but not other fatty acids, strikingly raised the expression of Heme Oxygenase-1 (HO-1) over 100 times in gingival epithelial cells [83]. HO-1 is a cytoprotective enzyme that takes part in heme catabolism and exhibits anti-oxidative function through biliverdin and CO [84]. An investigation confirmed that phosphorylation of ERK is an upstream signaling molecule that corresponds to the Nrf2-Antioxidant Response Element (ARE) activity in microglial cells [85]. Previous studies have reported that the elevation of the anti-oxidative molecule is mediated by the translocation of Nrf2 into the nucleus and binds to an ARE-promoter [86,87,88]. In this matter, KetoC has a unique molecular structure of an α, β-unsaturated carbonyl moiety consisting of a carbonyl group at position ten and a *trans*-double bond at position 11 of its chain [35], that these structures generate the activation of Nrf2-ARE signaling by binding to GPR efficiently [83]. In addition, the different molecular structure of fatty acid gives different affinities when it binds to the receptor [24], that eventually enhances its function. 

Furthermore, an in vivo study using a high-fat diet (HFD)-induced non-alcoholic steatohepatitis (NASH) mice model showed that 0.1% KetoC increased the level of high-density lipoprotein (HDL) related genes and HDL cholesterol levels in the plasma [41]. NASH is characterized by an injury, inflammation, oxidative stress, and fibrosis in the liver [89]. Furthermore, another in vivo study clearly described that KetoC treatment alone did not affect the abnormal bodyweight alteration, local, and systemic inflammation [21]. Collectively, these findings suggest that KetoC may partly affect the progression of NASH, an inflammatory disease in the liver, due to its beneficial modalities, without giving any adverse effects.

## 3. Conclusions

**Figure 2:**
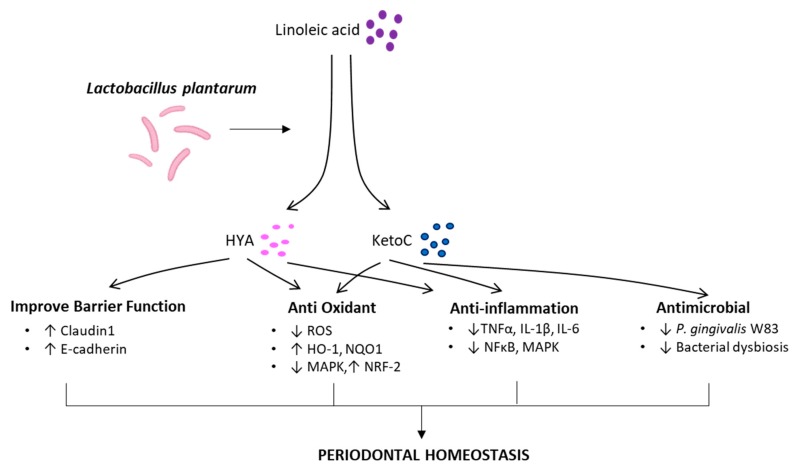
Beneficial functions of *Lactobacillus*-derived bioactive metabolites bring periodontal homeostasis. HYA and KetoC possess anti-oxidant [26,40], anti-inflammation [22,23,27,38,39,87], antimicrobial [21], and epithelial barrier junction improver [23,24].

In the present study, we propose a protective mechanism illustration of gut metabolites in regulating periodontal homeostasis (Figure 2). Based on supporting investigations, these findings may emphasize the connection between oral and gut bacteria in the equilibrium of the host defense system related to periodontal disease. Given the synergic effect of modulating oral and gut bacteria, this approach might potentially be implemented for a therapeutic target of periodontal disease. In the present review, *Lactobacillus*-derived fatty acids, KetoC and HYA, have multi-beneficial functions (anti-inflammatory, anti-oxidant, and antimicrobial) and bring great advantage for the development of therapeutic drugs. Currently, we expand the investigation of these metabolites for gut microbial modulators, as they may produce beneficial metabolic products in some ways. One can speculate that metabolome may improve systemic health. To the best of our knowledge, studies to conduct these metabolites have been limited to in vitro and in vivo studies. Further analyses, for example, the delivery system optimization of these metabolites, are required prior to the clinical research.

## Figures and Tables

**Table 1:** Studies of gut metabolite in relation to periodontal disease.

No	Author (Year)	Metabolite	Modality	Study	Outcome
1	Miyamoto et al. (2015) [24]	HYA	Improve epithelial barrier function	In vitro	HYA improves intestinal epithelial barrier impairment partially via GPR40-MEK-ERK pathway.
2	Furumoto et al. (2016) [26]	KetoC	Antioxidant	In vitro	KetoC increases antioxidant genes by upregulating the NRF2-ARE pathway in HepG2 cells.
3	Yang et al. (2017) [27]	KetoC	Anti-inflammatory	In vitro	KetoC exerts anti-inflammatory function via MAPK and NFκB signaling in macrophages induced with bacterial lipopolysaccharide.
4	Kaikiri et al. (2017) [38]	HYA	Anti-allergic and anti-inflammatory	In vivo	HYA feeding decreased TNF-α and increased claudin-1 (tight junction protein) levels in the mouse skin of atopic dermatitis (AD) model.
5	Yamada et al. (2018) [23]	HYA	Improve epithelial barrier function	In vivo	HYA tends to prevent alveolar bone loss in periodontitis model by improving the expression of E-cadherin in gingival tissue.
				In vitro	HYA increases beta defensin, thereby inhibiting inflammation
6	Ikeguchi et al. (2018) [39]	KetoC and HYA	Anti-inflammatory	In vitro	KetoC and HYA were found to inhibit ERK phosphorylation induced by LPS in microglial cells.
7	Sulijaya et al. (2018) [22]	KetoC	Anti-inflammatory	In vitro	GPR120 mediates the suppression function of KetoC towards TNFα in *P. gingivalis* LPS-induced inflammation through NfκB p65 pathway.
8	Sulijaya et al. (2019) [21]	KetoC	Antimicrobial	In vivo	Oral gavage of KetoC reduces alveolar bone loss in *P. gingivalis* W83-induced periodontitis mice model.
				In vitro	KetoC inhibits *P. gingivalis* strain W83 growth in a dose-dependent manner.
9	Takeuchi et al. (2020) [40]	KetoC	Antioxidant	In vitro	KetoC counters oxidative stress condition in gingival epithelial cells through GPR120-Nrf2 ARE-MAPK pathway.
10	Sofyana et al. (2020) [41]	KetoC	HDL modulator	In vivo	KetoC upregulates HDL related genes and HDL cholesterol levels in the plasma.
